# Strong but diverging clonality - climate relationships of different plant clades explain weak overall pattern across China

**DOI:** 10.1038/srep26850

**Published:** 2016-06-01

**Authors:** Duo Ye, Guofang Liu, Yao-Bin Song, William K. Cornwell, Ming Dong, Johannes H. C. Cornelissen

**Affiliations:** 1 Key Laboratory of Hangzhou City for Ecosystem Protection and Restoration, Hangzhou Normal University, Hangzhou, China; 2 College of Chemistry and Life Sciences, Zhejiang Normal University, Jinhua, China; 3 State Key Laboratory of Vegetation and Environmental Change, Institute of Botany, Chinese Academy of Sciences, Beijing, China; 4 Systems Ecology, Department of Ecological Science, Faculty of Earth and Life Sciences, VU University Amsterdam, The Netherlands; 5 Ecology and Evolution Research Centre, School of Biological, Earth and Environmental Science, University of New South Wales, Sydney, NSW 2052, Australia

## Abstract

The clonal strategy should be relatively important in stressful environments (i.e. of low resource availability or harsh climate), e.g. in cold habitats. However, our understanding of the distribution pattern of clonality along environmental gradients is still far from universal. The weakness and inconsistency of overall clonality-climate relationships across taxa, as reported in previous studies, may be due to different phylogenetic lineages having fundamental differences in functional traits other than clonality determining their climate response. Thus, in this study we compared the clonality-climate relationships along a latitudinal gradient within and between different lineages at several taxonomic levels, including four major angiosperm lineages (Magnoliidae, Monocotyledoneae, Superrosidae and Superasteridae), orders and families. To this aim we used a species clonality dataset for 4015 vascular plant species in 545 terrestrial communities across China. Our results revealed clear predictive patterns of clonality proportion in relation to environmental gradients for the predominant representatives of each of the taxonomic levels above, but the relationships differed in shape and strength between the 4 major angiosperm lineages, between the 12 orders and between the 12 families. These different relationships canceled out one another when all lineages at a certain taxonomic level were pooled. Our findings highlight the importance of explicitly accounting for the functional or taxonomic scale for studying variation in plant ecological strategy across environmental gradients.

Clonal growth, i.e. the production of potentially independent offspring by vegetative growth, gives plant species the ability to forage (by moving spacers to exploit favourable patches), integrate (by sharing resources among ramets) and store (by translocating resources into clonal growth organs)^1^. The clonal strategy should be relatively important in stressful environments, e.g. in cold, dry or particularly wet habitats. This means that clonality, defined here as the proportion of species in the community capable of being clonal, should be common in more stressful conditions^2^. However, while some studies found some empirical evidence for this^3^,^4^,^5^, other studies did not^6^.

Here we argue that the lack of consistency of overall clonality-climate relationship may be due to issues of scale; not only spatial scale^2^,^6^, but also functional scale, as determined by the traits of different plant functional types or major clades. Some of the traits that differ between lineages may be influential in the plants’ responses to climatic variation, independently of clonality. Other traits may constrain the evolutionary possibilities of developing or losing clonal organs. Thus, within species groupings, phylogenetic conservatism, i.e. constraints inherited from the evolutionary history of their traits^7^ will determine whether there is much evolutionary potential for divergence of clonal versus non-clonal habit across climatic gradients. Indeed, clonality, while occurring in multiple, phylogenetically wide-ranging lineages^3^,^8^, takes very different forms and functions in different clades, some of which may be constrained differently in their responses to environmental gradients^9^,^10^,^11^.

In a recent study, Ye *et al.*^2^ compared proportions of clonal plant species within communities along large climatic gradients across China, i.e. from the cold temperate zone to the tropics. They found that overall site-level proportion of clonality, among all vascular species present, had a significant albeit weak positive relationship with climate harshness (i.e., towards colder or drier parts of the gradient). However, they also found the relationships between proportion of clonal species and climatic variables (mean annual temperature, mean annual precipitation, temperature seasonality and precipitation seasonality) to be weak. Here we argue that this lack of clear overall pattern may be due to the fact that all species were treated equally in their study. If the relationship is strong in some species groups but very weak or even opposite in some other groups, the resultant overall relationship would still be weak. Therefore we hypothesize that clearer climate-clonality relationships should emerge when the species are analyzed at a finer specific functional and taxonomic scale, as determined by the traits of different clades. There should be different clonality-climate relationships for different clades. Specifically, we formulate two predictions: 1) clades which are likely to have had clonality as the ancestral state, and which are still predominantly clonal, might not show a clear response to climatic gradients; 2) some basal clades which originated as non-clonal in tropical regions might be more likely to evolve clonality for tolerating colder or drier climate when they spread along the latitudinal gradient. We test our hypotheses by comparing clonality-climate relationships among predominant representatives at three taxonomic levels, i.e. between four major angiosperm lineages, 12 orders and 12 families respectively using a clonality dataset for 4015 vascular plant species in 545 terrestrial communities across the whole of China.

## Results

### Variation of clonality between clades

Clonal plants occurred in many clades (Table 1). Within each taxonomic level, significant variation was observed among its component members (all *P *< 0.001). Among four major angiosperm lineages, proportions of clonality was higher in Monocotyledoneae (Table 1); at order level, Polypodiales and Poales had higher proportions with Ericales being intermediate and Laurales conspicuously poor in clonality (Table 1). At family level, proportions of clonality in Poaceae and Cyperaceae were much higher than in other clades, followed by Ericaceae and Primulaceae, while Lauraceae and Pentaphyllaceae were almost exclusively non-clonal (Table 1). Note that, apart from Polypodiales (ferns), there is strong nestedness of proportion clonality between taxonomic levels.

### Variation of latitudinal pattern of clonality within different clades

**Figure 1:**
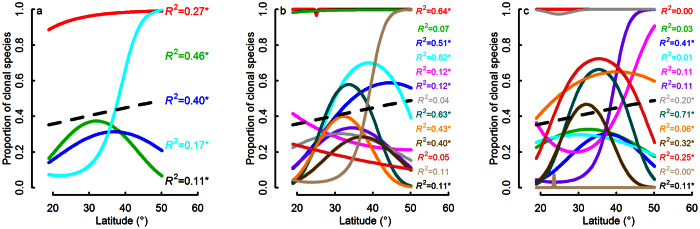
The differences of clonality - latitude relationships at different taxonomic levels. Resultant overall clonality - latitude relationship (dashed black line, *R*^*2*^ = 0.11) was weak because different underlying relationships canceled each other out. The sequence of *R*^*2*^ in the figure corresponds with the overall proportion of clonal species of each clade. a: for major four angiosperm lineages (red line: Monocotyledonae, *R*^2^ = 0.27; green line: Superasteridae, *R*^2^ = 0.46; blue line: Superrosidae, *R*^2^ = 0.40; cyanic line: Magnoliidae, *R*^2^ = 0.17) b: for 12 orders (red: Polypodiales, *R*^2^ = 0.64; green: Poales, *R*^2^ = 0.07; blue: Ericales, *R*^2^ = 0.51; cyanic: Malpighiales, *R*^2^ = 0.62; magenta: Rosales, *R*^2^ = 0.12; purple: Lamiales, *R*^2^ = 0.12; gray: Asterales, *R*^2^ = 0.04; dark cyanic: Gentianales, *R*^2^ = 0.63; orange: Sapindales, *R*^2^ = 43; dark goldenrod: Fabales, *R*^2^ = 0.40; brown: Fagales, *R*^2^ = 0.05; bisque: Laurales, *R*^2^ = 0.11) c: for 12 families (red: Poaceae, *R*^2^ = 0.00; green: Asteraceae, *R*^2^ = 0.03; blue: Fabaceae, *R*^2^ = 0.41; cyanic: Rosaceae, *R*^2^ = 0.01; magenta: Fagaceae, *R*^2^ = 0.11; purple: Lauraceae, *R*^2^ = 0.11; gray: Cyperaceae, *R*^2^ = 0.20; dark cyanic: Rubiaceae, *R*^2^ = 0.71; orange: Ericaceae, *R*^2^ = 0.06; dark goldenrod: Lamiaceae, *R*^2^ = 0.32; brown: Primulaceae, *R*^2^ = 0.25; bisque: Pentaphylacaceae, *R*^2^ = 0.00).

Among four major lineages, proportions of clonal species in Magnoliidae and Monocotyledoneae increased with increasing latitude, while in Superrosidae and Superasteridae had a hump-back relation with latitude (Fig. 1a). At order level and family level, a mixture of latitudinal patterns was found among different clades (Fig. 1b,c). Supplementary figures S1, S2 and S3 show the actual spatial patterning of proportion clonality on the map of China at each taxonomic scale. These maps serve to visualize the above relationships with latitude as well as some of the climate relationships reported below.

### Variation of climatic pattern of clonality within different clades

**Figure 2:**
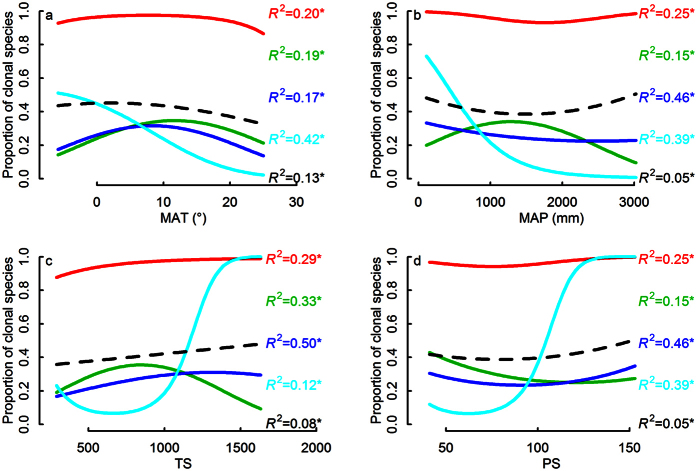
The differences of clonality - climate relationships among major four angiosperm lineages. Resultant overall clonality - climatic relationship (dashed black line) was weak because different underlying relationships for Monocotyledonae (red line), Magnoliidae (cyanic line), Superrosidae (blue line) and Superasteridae (green line) canceled each other out. The sequence of *R*^2^ in the figure corresponds with the overall proportion of clonal species of each clade.

**Figure 3:**
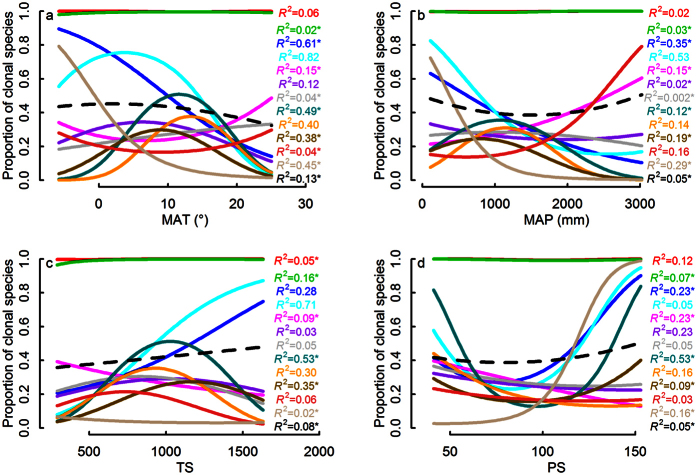
Clonality - climate relationships at order level. Resultant overall clonality - climate relationship (dashed black line) was weak because different underlying relationships for 12 orders (red: Polypodiales; green: Poales; blue: Ericales; cyanic: Malpighiales; magenta: Rosales; purple: Lamiales; gray: Asterales; dark cyanic: Gentianales; orange: Sapindales; dark goldenrod: Fabales; brown: Fagales; bisque: Laurales) canceled each other out. The sequence of *R*^2^ in the figure corresponds with the overall proportion of clonal species of each clade.

**Figure 4:**
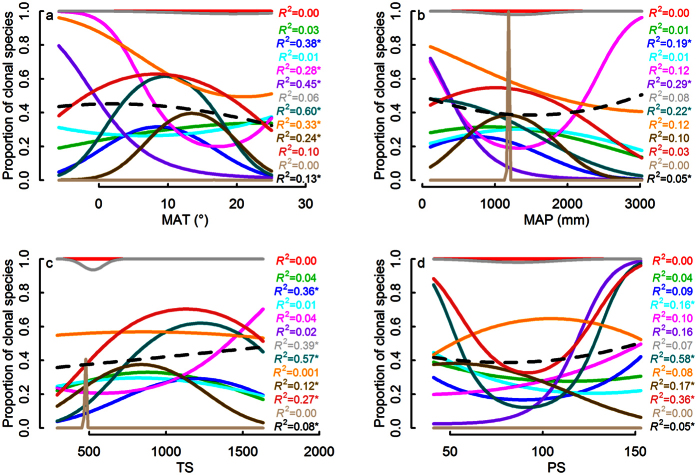
Clonality - climate relationships at family level. Resultant overall clonality - climate relationship (dashed black line) was weak because different underlying relationships for 12 families (red: Poaceae; green: Asteraceae; blue: Fabaceae; cyanic: Rosaceae; magenta: Fagaceae; purple: Lauraceae; gray: Cyperaceae; dark cyanic: Rubiaceae; orange: Ericaceae; dark goldenrod: Lamiaceae; brown: Primulaceae; bisque: Pentaphylacaceae) canceled each other out. The sequence of *R*^2^ in the figure corresponds with the overall proportion of clonal species of each clade.

Logistic regressions showed that there was a variety of strengths and shapes of clonality-climate relationships among clades at the three taxonomic levels (Figs 2, 3, 4). Proportion of clonal species of Magnoliidae, Monocotyledoneae, Ericales, Laurales, Malpighiales, Ericaceae, Fagaceae and Lauraceae decreased with increasing MAT, but proportion of clonal species of Rosales showed the opposite pattern; Proportion of clonal species of Superrosidae and Superasteridae, Fabales, Gentianales, Sapindales, Lamiaceae, Rubiaceae and Fabaceae had a hump-back relation with MAT; other clades showed no relationship between clonality and MAT (Figs 2a, 3a and 4a).

Similarly, the relationship between clonality and MAP, TS or PS showed a striking mixture of different linear and curvilinear relationships (of different strengths), in several cases even opposite patterns within different taxonomic levels (Figs 2, 3, 4). For instance, Rosales and Fagales had a positive relation with MAP, while Malpighiales had a strong negative relationship (Fig. 3b). For temperature seasonality (TS) Superasteridae had a hump-back relation while Magnoliidae had an upside-down hump-back (Fig. 4c). For precipitation seasonality (PS) Rubiaceae had an upside-down hump-back while Lamiaceae and Rosaceae had a weak downward curve (Fig. 4d).

## Discussion

The ability of plants to grow clonally has long been considered to be important in stressful environments. Previous studies have attributed convergence of plant traits to a filter effect of environmental factors^12^,^13^, i.e., clonal plants tend to pass the climatic stress filter^14^ successfully in cold or wet environments. However, the environmental gradient patterns of clonality have not been confirmed to be as universal as expected^6^,^10^,^15^. Ye *et al.*^2^ also found that the relationship between proportion of clonal species and climatic variables, while statistically significant, was weak in a large dataset from across China. Based on our new findings here, we can now conclude that this weakness of pattern was because different underlying relationships for different phylogenetic lineages canceled each other out when the entire species set was pooled without taking phylogeny into account. Indeed, the striking mixture of different strengths and shapes of relationships between proportion clonality and four main macroclimatic variables support our main hypothesis.

Previous comparative studies on the flora of central Europe showed that clonal plants occurred in many clades^3^. Our results are consistent with those studies. Furthermore, we found that clades with more herbaceous species (i.e. Monocotyledonae and ferns belonging to Polypodiales) included more clonal species, while clades with more woody species (i.e. Fagales and Laurales) included fewer clonal members. This is consistent with a previous report that trees are less often clonal than herbs^16^.

Our results, in general, provide more support for the first specific hypothesis. Clades like Monocotyledonae, as well as Poales, Poaceae, Cyperaceae, which are likely to have had clonality as the ancestral state, and which are still predominantly clonal, showed no clear response to climatic gradients. This suggests that the clonality distribution of grasses and sedges and their allies cannot be explained by the ‘phylogenetic niche conservatism’ hypothesis. Humphreys *et al.*^17^ also found that temperature (low temperature in particular) appears not to limit the distribution of this clade.

In support of our main hypothesis we found phylogenetic variation in clonality-climate relationships at given coarser levels of taxonomy along our large latitudinal gradient. With the refinement of the lineages, we found that there were more diverse response strategies of clonal species to climate. What underlies the large differences in clonality-climate relationship among different clades? The ancestors of extant species of a given clade were adapted to particular climatic regimes through trait evolution. The trait heritage of this deep-time evolution observed in extant species, called phylogenetic conservation, must be a plausible explanation for related species sharing similar traits in a particular climate belt^13^,^18^,^19^. Indeed, variation in functional traits has been linked to the different strategies and fitnesses of plant species in response to climatic variation or change^20^,^21^. Kimes *et al.*^22^ found that different clonal growth forms showed various trends of proportion clonality along the altitudinal gradient in Eastern Ladakh, Western Himalayas; non-spreading integrators prevailed on shaded rocky slopes, non-spreading splitters in wet grasslands and spreading splitters at the wettest sites. Evette *et al.* also found that the distribution of clonal traits was significantly but weakly correlated with altitude and duration of snow cover^23^. Several other studies found that different clonal traits underpinned different adaptive strategies with respect to environmental factors^10^,^15^,^24^. This indicates that different clonality-climate relationship may actually be due to different advantages brought by different clonal growth forms or organs. Consequently, some widely distributed clades such as Poales (or within them Poaceae and Cyperaceae) and Polypodiales could evolve more, and particularly successful, clonal growth forms, possibly also aided by evolution of other adaptive traits in response to climatic drivers. A strong dominance of clonality over non-clonal growth in such clades might thus enable clonal plants to cover a broader climatic envelope, which would result in weaker patterning of clonality with latitudinal or climatic gradients. Indeed, those clades dominated by herbaceous plants with high proportions of clonality, such as Monocotyledoneae, Poales, Asterales, Polypodiales, Poaceae, Cyperaceae, Primulaceae and Asteraceae, showed no significant relationship with climate.

Clonality-climate relationships also differ among the large clades with different evolutionary histories, i.e., ancestral climatic niches of different origins. There is increasing consensus that niche conservatism might play an important role in shaping species richness–climate relationships along latitudinal gradients^25^,^26^. For instance, in agreement with our second specific prediction, clades like Magnoliidae, Lauraceae (including Laurales) and Fagales, which originated in tropical regions or under ancient warm climatic conditions^27^,^28^, had an increasing proportion of clonal species with increasing latitude along a contemporary climatic gradient.

In summary, our results support our original hypothesis. Clonality-climate relationships vary greatly in both shape and strength among higher taxa at the same taxonomic ‘height’ in the Tree of Life. These different patterns for different lineages cancel each other out in the complete species set, resulting in poor climate-clonality relationships across all vascular plant species together. The general message from our study is that much important ecological and evolutionary information is missed when climate-trait relationships are not considered in sufficient detail. Even (or especially) to find ‘the big picture’, it is necessary to take into consideration different functional or taxonomic levels to test the variation in plant ecological strategy across large spatial scale or environmental gradients.

## Material and Methods

### Plant species pool

Full methodological details of data collecting are in Ye *et al.*^2^. In brief, vegetation composition data for a total of 545 communities across China was collected, ranging from 18°50′24″ to 50°7′48″N, and from 80°26′24″ to 128°4′12″E. The vegetation surveys followed standard methodology^29^, with a spatial nesting design. For forest communities, plant species in the tree, shrub, and herb layers were recorded in one 20 × 20 m plot, four 2 × 2 m plots and four 1 × 1 m plots, respectively. In the shrub communities, the 20 × 20 m plot was left out, while in the herbaceous communities, only plants in the four 1 m × 1 m plots were recorded. In total, there were 4015 species (227 families and 72 orders) in 545 plots, of which 1579 clonal species (154 families and 59 orders) were determined. Clades that appeared more frequently at each of three taxonomic levels were chosen in this study: four major angiosperm clades (Magnoliidae, Monocotyledoneae, Superrosidae and Superasteridae), 12 orders (Poales, Ericales, Rosales, Asterales, Fabales, Fagales, Laurales, Malpighiales, Polypodiales, Gentianales, Lamiales and Sapindales) and 12 families (Poaceae, Cyperaceae, Ericaceae, Primulaceae, Rosaceae, Asteraceae, Lamiaceae, Rubiaceae, Fagaceae, Fabaceae, Lauraceae and Pentaphylacaceae).

### Identification of plant clonality

According to the clonality descriptions in the book ‘Flora Reipublicae Popularis Sinicae’^30^, all vascular species in this research were categorized into non-clonal plants and clonal plants. Moreover, the CLO-PLA database^31^ was used for plant clonality identification based on the 17 clonal growth organs described there: (1) Rooting horizontal stems at or above soil surface; (2) turions; (3) bulbils and tubers of stem origin at or above soil surface; (4) plantlets (pseudo-vivipary); (5) plant fragments of stem origin; (6) budding plants; (7) root tubers at or above soil surface; (8) buds on leaves (gemmipary); (9) epigeogenous rhizomes; (10) hypogeogenous rhizomes; (11) tuber-splitters; (12) stem tubers; (13) bulbs; (14) root-splitters; (15) adventitious buds on roots; (16) root tubers belowground; (17) offspring tubers at distal end of aboveground stems.

### Climate data

For each site, four climate variables related to temperature and moisture regimes were derived from the WorldClim database^32^: mean annual temperature (MAT, °C), mean annual precipitation (MAP, mm), temperature seasonality (TS) and precipitation seasonality (PS). MAT and MAP represent the annual average trends of temperature and moisture for a given site, respectively. Seasonality (TS and PS) is defined as the standard deviation of the weekly precipitation, expressed as a percentage of the annual mean (MAT or MAP), representing intra-annual climate instability.

### Data analysis

The proportion of clonal species in a plant community within each clade, representing the preponderance of clonality, was determined by dividing the number of clonal species by the total number of species of the given clade in a plant community.

Because the proportion data (clonal versus non-clonal) obeys a binomial distribution, logistic regressions (one of the generalized linear models) were used to test the relationships between the proportion of clonal species and each climatic variable. In these regressions, we weighted the proportion of clonal species with species richness in each plot so as to minimize the influence of chance effects for sites with small sample size for clonal proportions on the overall patterns. All the analyses were performed in R software 3.1.2^33^.

## Additional Information

**How to cite this article**: Ye, D. *et al.* Strong but diverging clonality - climate relationships of different plant clades explain weak overall pattern across China. *Sci. Rep.* **6**, 26850; doi: 10.1038/srep26850 (2016).

## Supplementary Material

Supplementary Information

## Figures and Tables

**Table 1:** Variations of proportion of clonal species in clades at different taxonomic levels.

Major lineages	Mean	SD		Order	Mean	SD		Family	Mean	SD	
Monocotyledonae	0.96	0.08	a	Polypodiales	1.00	0.01	a	Poaceae	1.00	0.00	a
Superasteridae	0.31	0.18	b	Poales	0.99	0.03	a	Cyperaceae	0.99	0.06	a
Superrosidae	0.25	0.18	c	Ericales	0.41	0.33	b	Ericaceae	0.62	0.34	b
Magnoliidae	0.17	0.28	d	Malpighiales	0.38	0.38	bc	Primulaceae	0.55	0.41	b
				Rosales	0.34	0.3	bc	Rosaceae	0.33	0.34	c
				Lamiales	0.28	0.31	cd	Asteraceae	0.30	0.28	c
				Asterales	0.27	0.27	cd	Lamiaceae	0.28	0.33	cd
				Gentianales	0.27	0.35	cd	Rubiaceae	0.27	0.36	cd
				Sapindales	0.27	0.32	cde	Fagaceae	0.23	0.31	cd
				Fabales	0.17	0.23	de	Fabaceae	0.18	0.24	de
				Fagales	0.14	0.19	ef	Lauraceae	0.08	0.19	ef
				Laurales	0.07	0.19	f	Pentaphylacaceae	0.00	0.02	f

Means and standard deviations (SD) are given. Pairs of means with lowercase letters that do not overlap indicate the means being significantly different at *P* = 0.05.
