# Supplementary material for: Strong but diverging clonality - climate relationships of different plant clades explain weak overall pattern across China
Source: Sci Rep. 2016 Jun 1;6:26850. doi: 10.1038/srep26850 (PMC4887789; doi:10.1038/srep26850)
Supplement: Supplementary Information [file srep26850-s1.pdf]

Strong but diverging clonality - climate relationships of different plant clades explain weak overall pattern across China

Duo Ye[1,2,4], Guofang Liu[4], Yao-Bin Song[1], William K. Cornwell[3,5], Ming Dong[1,4,*], Johannes H.C. Cornelissen[3]

* *For correspondence. E-mail: dongming@hznu.edu.cn*

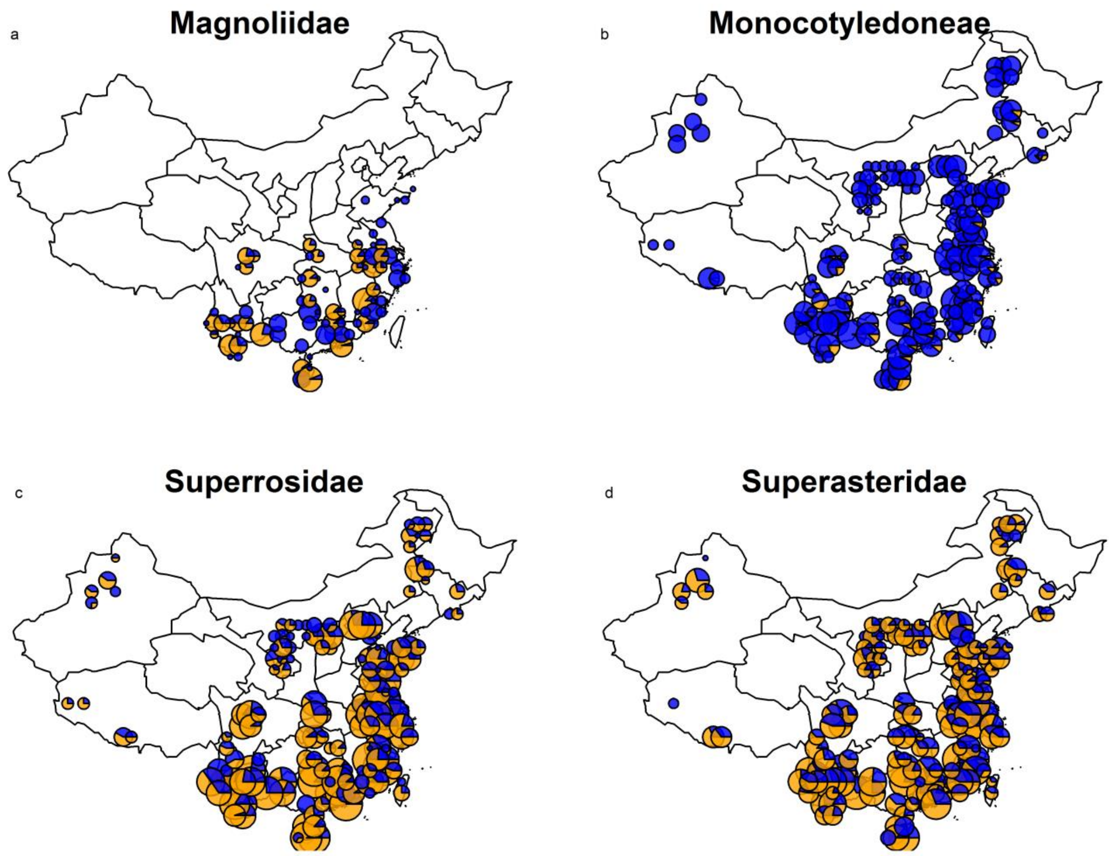

Supplementary Figure S1 Distribution pattern of clonality within 4 major clades along latitudinal gradients across China. The pie charts comprise blue and orange sectors denoting the relative proportions of clonal and nonclonal plants, respectively in a site. All the maps in supplementary figures were created by means of the *map* function of the *maps* and *mapdata* package in R software 3.1.2 (R Core Team 2014, https://www.r-project.org/).

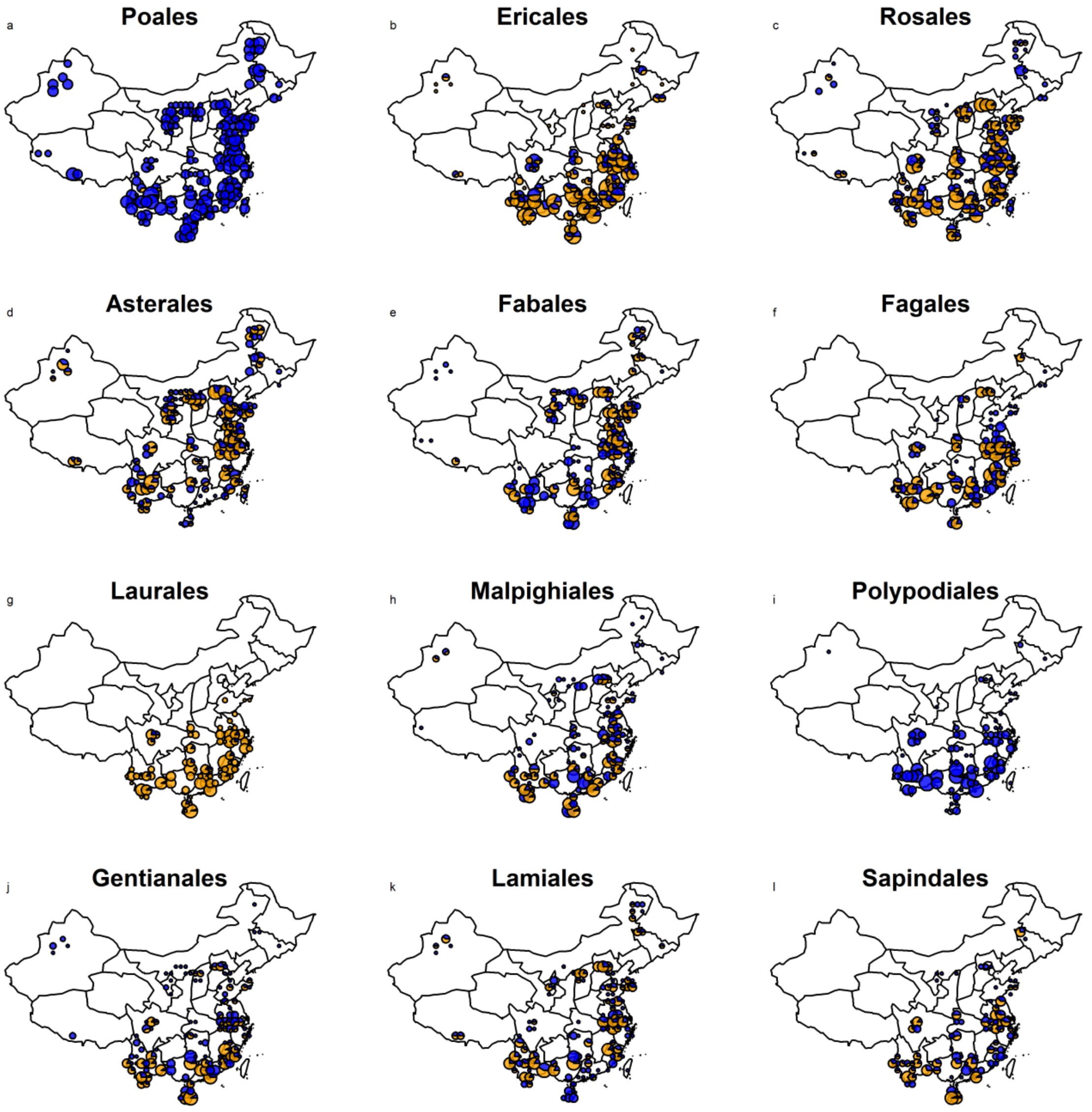

Supplementary Figure S2 Distribution pattern of clonality within 12 orders along latitudinal gradients across China. The pie charts comprise blue and orange sectors denoting the relative proportions of clonal and nonclonal plants, respectively in a site.

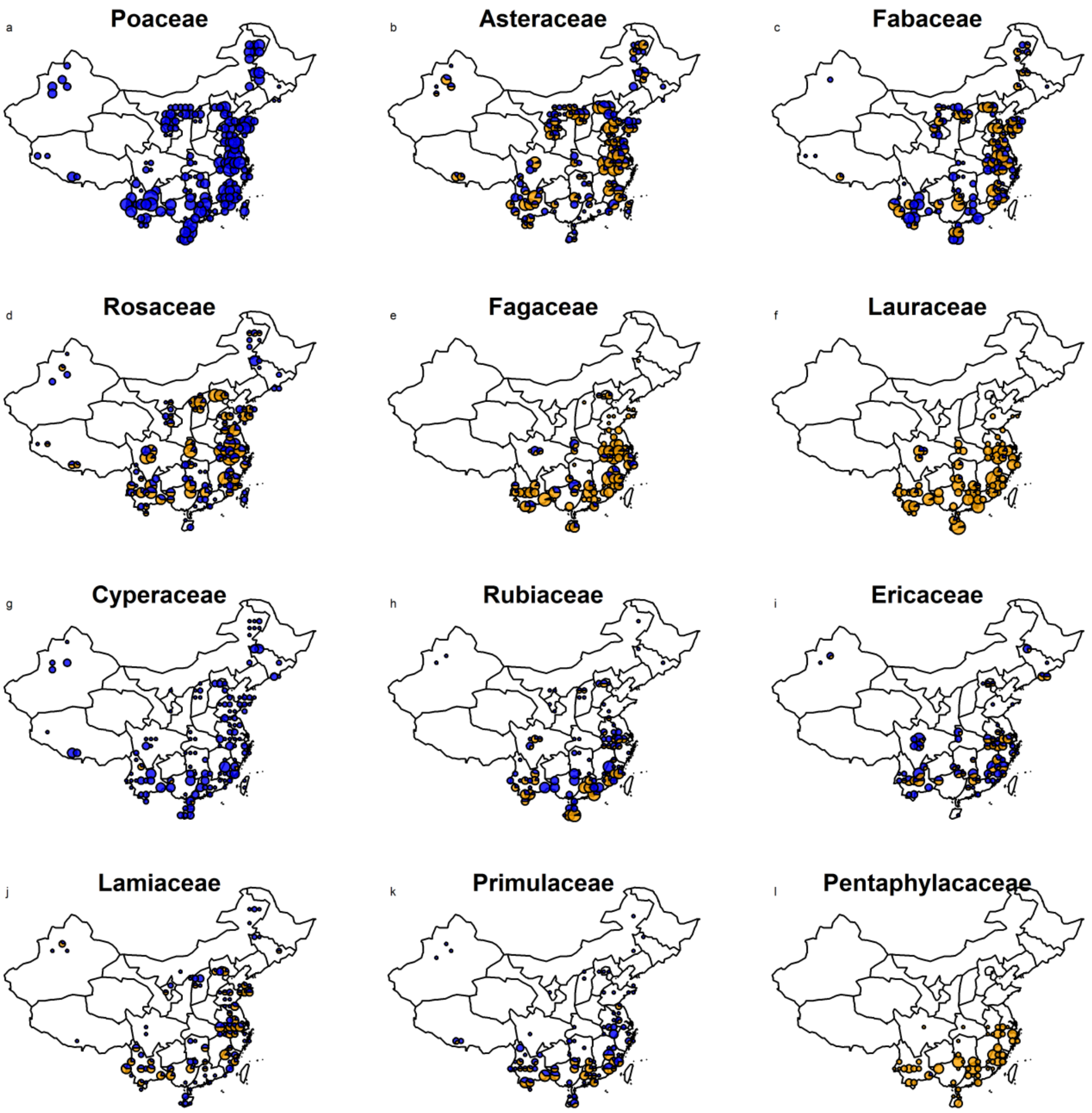

Supplementary Figure S3 Distribution pattern of clonality within 12 families along latitudinal gradients across China. The pie charts comprise blue and orange sectors denoting the relative proportions of clonal and nonclonal plants, respectively in a site.
